# Examining Anxiety Treatment Information Needs: Web-Based Survey Study

**DOI:** 10.2196/31338

**Published:** 2022-05-12

**Authors:** Matthew T Bernstein, Kristin A Reynolds, Lorna S Jakobson, Brenda M Stoesz, Gillian M Alcolado, Patricia Furer

**Affiliations:** 1 Department of Psychology University of Manitoba Winnipeg, MB Canada; 2 Centre for the Advancement of Teaching and Learning University of Manitoba Winnipeg, MB Canada; 3 Department of Clinical Health Psychology University of Manitoba Winnipeg, MB Canada

**Keywords:** anxiety, information needs, anxiety treatment, web survey, survey methodology

## Abstract

**Background:**

Several treatments for anxiety are available, which can make treatment decisions difficult. Resources are often produced with limited knowledge of what information is of interest to consumers. This is a problem because there is limited understanding of what people want to know when considering help for anxiety.

**Objective:**

This study aimed to examine the information needs and preferences concerning treatment options for anxiety by assessing the following: what information people consider to be important when they are considering treatment options for anxiety, what information people have received on psychological and medication treatment in the past, how they received this information in the past, and whether there are any differences in information needs between specific samples and demographic groups.

**Methods:**

Using a web-based survey, we recruited participants from a peer-support association website (n=288) and clinic samples (psychology, n=113; psychiatry, n=64).

**Results:**

Participants in all samples wanted information on a broad range of topics pertaining to anxiety treatment. However, they reported that they did not receive the desired amount of information. Participants in the clinic samples rated the importance of information topics higher than did those in the self-help sample. When considering the anxiety treatment information received in the past, most respondents indicated receiving information from informational websites, family doctors, and mental health practitioners. In terms of what respondents want to learn about, high ratings of importance were given to topics concerning treatment effectiveness, how it works, advantages and disadvantages, what happens when it stops, and common side effects.

**Conclusions:**

It is challenging for individuals to obtain anxiety-related information on the range of topics they desire through currently available information sources. It is also difficult to provide comprehensive information during typical clinical visits. Providing evidence-based information on the web and in a brochure format may help consumers make informed choices and support the advice provided by health professionals.

## Introduction

### Background

Anxiety disorders are one of the most common classes of mental health problems in the community [1]. Several treatment options are available for anxiety, which can make treatment decisions difficult for consumers. A way to support the treatment decision-making process is by providing high-quality information [2]. However, information about many health treatments (including treatment for anxiety) is seldom addressed and is difficult to access using currently available resources [3]. The internet is an important source of support, especially with the recent COVID-19 pandemic [4]. Unfortunately, available information on the internet tends to focus on descriptions of health problems and treatment options and provides little research-based evaluation of treatment options [5]. Professionals commonly produce resources for the public with limited knowledge of what information is of interest to consumers [3]. Indeed, existing resources often focus on a narrow range of options (ie, one or two) while overlooking others [5,6]. Making a wide range of information available to those seeking treatment for a health problem is an important step that can be taken to support treatment decision-making in individuals with different information needs and preferences.

There is a limited understanding of what people want to know when considering help for anxiety disorders, which is problematic because many people have unanswered questions not covered by currently available materials [7-9]. A recent systematic review of 12 studies on information and decision-making needs for mental health problems, such as depression and schizophrenia, revealed that *basic facts*, *treatment*, and *coping* were the most frequently cited information needs [10]. Tlach et al [10] emphasized the importance of discussing these topics with one’s health care provider to gather information and make informed decisions. Liebherz et al [3] conducted a web-based study of a German sample of individuals with anxiety disorders that examined patients’ information and decision-making needs and how they might inform the development of patient decision aids. These authors found that individuals with anxiety disorders reported receiving insufficient information from health care providers. Previous work by our research group has explored information needs and preferences concerning treatment options for depression, anxiety, and stress in young adults from community and college samples [7-9]. Our findings suggested that people dealing with these mental health issues want information on a broad range of topics to support their treatment decisions. Cunningham et al [8] also reported that individuals differ widely in the information they want and how they prefer to receive this information. By determining information preferences, health care providers can understand how to best deliver information to consumers.

### Objectives

This study addresses the following gap in the literature: there is a limited understanding of what persons with anxiety want to know about anxiety treatment. Furthermore, increasing health care providers’ understanding of patient information needs will enhance the shared decision-making process. In this study, we evaluated the information needs of adults (aged ≥18 years) seeking support and treatment information for problems with anxiety. We built on earlier research exploring information needs by our research group by recruiting individuals seeking information on the web or from mental health treatment clinics and asking questions about the amount of information individuals had received on different topics. Our goal was to examine the following questions: (1) What information do people consider to be important when they are considering treatment options for anxiety? (2) What information have people received on psychological and medication treatment in the past? (3) How did they receive this information in the past? (4) Are there any differences in information needs between specific samples and demographic groups?

## Methods

### Participants and Procedures

#### Clinic Recruitment

Individuals referred by their family physician for anxiety problems to either a hospital-based anxiety clinic offered through psychology or a hospital-based psychiatric consultation service were invited to participate. Participants recruited from each clinic (before treatment) were provided with information explaining the study’s procedures and a URL address they could use to access and complete a web-based survey. The response rates for the psychology and psychiatry samples were 23.2% (113/487) and 21.3% (64/300), respectively.

#### Website Recruitment

To provide a comparison with those seeking treatment for anxiety in hospital-based clinics, we also recruited a *self-help* sample from visitors to the Anxiety Disorders Association of Manitoba (ADAM) website (a local peer-support association). This website is widely visited by public members searching for information concerning anxiety disorders and treatment or peer-support services provided by the ADAM. Typically, more than 2000 visitors visit the ADAM website per month. A link to the survey was posted on the ADAM website, inviting interested people to click on a link to the survey.

The web-based consent form was the first webpage viewed by the participants when they visited the survey URL address. The consent form described the study’s purpose and highlighted that the choice to participate would not have an impact on the care they received from the treatment settings. Participants were asked to click “yes, I consent” (and then taken to the survey) or “no, I do not consent” (asked to close the browser).

### Ethics Approval

This study was approved by the University of Manitoba Psychology and Sociology Research Ethics Board (protocol 2018:011) and St Boniface Hospital Research Review Committee (RRC/2018/1753).

### Measures

#### Information Needs Questions

Many of the questions in this section of the survey were adapted from previous research by our group on information needs and preferences concerning mental health issues [7,9], allowing for the replication and extension of this work. Participants were first asked, “If you were having anxiety problems and considering getting help, what information would be important to you in considering the kinds of help available?” They were presented with a list of 20 information topics, including treatment options (eg, medication and psychological treatments) and information related to treatment, such as cost and side effects. These topics were rated on a scale from 0 (*not at all important*) to 8 (*very important*). Following these questions, participants were asked if they had previously received psychological treatment for anxiety. If they answered yes, they were presented with 12 information topics and asked what specific information they had received in the past regarding psychological treatment, along with an additional question regarding the information received about the use of medication. These questions were rated on a scale ranging from 0 (*none*) to 8 (*just the right amount*). If they answered no, they skipped to the next set of questions, which addressed whether participants had previously received medication treatment for anxiety problems. If they answered yes to this question, they were asked which of the 13 information topics regarding medication treatment they had received information about in the past (eg, cost and how long it takes to produce results), along with an additional question about the amount of counseling or therapy information they had received. These questions were rated on a scale ranging from 0 (*none*) to 8 (*just the right amount*). If they answered no, they skipped to the final section, where they were asked, “When you have been considering treatment options for anxiety in the PAST, how much information did you receive from each of following sources?” The 10 items in this section were rated on a scale from 0 (*none*) to 8 (*a lot*).

#### Sample Characteristics

Participants were asked to provide information concerning their gender, age, marital status, education level (ie, sum of years of education in high school, college, university, and apprenticeship categories), the main activity in the past 12 months (ie, work and school), and country of birth. In addition, they were asked if they had previously been diagnosed with an anxiety disorder by a health care professional. They were also asked if they had previously received psychological or medication treatment or if there was a time when they felt they would have benefited from either treatment but did not receive it. Finally, they were asked about their experience with self-help approaches (eg, exercise and meditation).

#### Anxiety Symptoms

Participants’ current level of anxiety symptoms was assessed using the PROMIS (Patient-Reported Outcomes Measurement Information System) Anxiety Scale (short form), which is a validated measure of anxiety symptoms [11]. The survey uses the following introductory statement: “In the past 7 days...” This is followed by 8 items rated on a 5-point rating scale ranging from 1 (*never*) to 5 (*always*). This scale has good internal consistency with a Cronbach α of .93 reported in previous work [11], similar to the internal consistency found for these items in this study (Cronbach α=.92). This scale also has good validity (divergent, *r*=0.72, and convergent, *r*=0.80).

### Statistical Analysis

#### Overview

We computed and tabulated descriptive statistics for sociodemographic variables and responses to questions about information experiences and preferences. Sociodemographic data obtained from different groups of respondents were compared using 1-way ANOVA tests for means and chi-square tests for proportions. CIs for mean ratings on the survey items were reported, allowing for convenient comparisons within and across different survey questions and groups of respondents.

In addition, we computed a composite *information importance* score, which reflected the number of topics receiving a high rating (≥6) for topic importance, and 2 composite *information received* scores, which reflected the number of topics pertaining to counseling or therapy or medication treatment that participants felt they had received their desired (or *appropriate*) amount of information in the past (rating≥6 for the amount of information received). This cutoff of ≥6 was selected, as the ratings were on a 9-point scale from 0 (*not at all important*) to 8 (*very important*). Therefore, a rating of 6 through 8 was considered a high rating of importance and suggested a strong interest in receiving this type of information. These composite scores were used as outcome variables in forced-entry multiple linear regression analyses that included the following predictors: gender, age, birthplace, marital status, education level, anxiety symptoms, anxiety diagnosis, and treatment experience. We ran separate regressions for the self-help and (combined) clinic samples, given that, as outlined in the *Sample Characteristics* Results section*,* the self-help group differed from the clinic samples on a variety of measures. The regressions conducted on the combined clinic sample included an additional predictor, namely, whether the individual was recruited from the psychology or psychiatric clinic.

#### Power

Before data collection, we conducted an a priori power analysis to determine the sample size required for a power of 0.80, a significance level of .05, and an effect size of Cohen *d*=0.50. The analysis yielded an intended sample size of 102. The medium effect size was selected based on the Cochrane collaboration review of the effects of decision aids for the treatment of health issues [12]. The goal of this study was to enroll a sample of 100 from each clinical group and, for comparison, enroll 200 nonclinical participants. Given that there was some difficulty enrolling participants from the psychiatry sample, we conducted a sensitivity power analysis after data collection. These analyses were on the ANOVAs, used to compare the demographic characteristics, and on the regressions, used to predict information importance sum scores, to determine the effect detectable given the sample sizes included in the study analyses. For the ANOVA, using a power of 0.80, a significance level of .05, and a total sample size of 465 led to a detectable effect size of Cohen *d*=0.14. For the self-help regression analyses, using a power of 0.80, a significance level of.05, and a self-help sample size of 283 led to a detectable effect size of Cohen *d*=0.06. For the clinic regression, analyses using a power of 0.80, a significance level of .05, and a clinic sample size of 170 led to a detectable effect size of Cohen *d*=0.10.

## Results

### Sample Characteristics

Most participants in all 3 groups were Canadian born. Most had previously been diagnosed with an anxiety disorder, and most reported that they felt they could have benefited from counseling or therapy from a professional for anxiety in the past but had not received it. Despite these similarities, there were several differences in the sample characteristics, particularly between the self-help sample and the 2 clinic samples (Table 1). The mean age of the self-help sample (32.2, SD 9.0 years; range 18-77 years) was significantly lower than that of the clinic samples (mean_psychology_ 38.2, SD 13.9 years; range 18-80 years; mean_psychiatry_ 37.6, SD 14.9 years; range 18-65 years; *F*_2,458_=14.8; *P*<.001; *η*_p_^2^=0.06). Compared with the clinic samples, the self-help sample also had the highest proportion of men (138/288, 47.9%; *χ*^2^_2_=14.4; *P*=.001), had more individuals who reported being married (*χ^2^*_2_=22.6; *P*<.001), were more likely to have been working full-time in the year before completing the survey (*χ^2^*_2_=55.0; *P*<.001), and reported more years of education (an average of 5 years after high school, compared with 2 years in the clinic samples; *F*_2,458_=32.1; *P*<.001; *η*_p_^2^=0.13). Compared with the clinic samples, a higher proportion of the self-help sample also indicated that there was a time when they felt that medication for anxiety would have been helpful, but they did not receive it (*χ*^2^_4_=24.9; *P*<.001). Both the self-help and psychology samples reported more symptoms of anxiety (a PROMIS *T* score >50) compared with the psychiatry sample (*χ*^2^_4_=8.3; *P*=.02). Finally, compared with the self-help and psychiatry samples, a higher proportion of the psychology sample reported that they had received counseling or therapy (*χ*^2^_4_=10.9; *P*=.004) and medication (*χ*^2^_4_=10.7; *P*=.005) for anxiety in the past.

**Table 1:** Sociodemographic characteristics of survey respondents.^a^

	Self-help sample (n=288)	Psychology sample (n=113)	Psychiatry sample (n=64)	*P* value
Age (years), mean (SD)	*32.2* (*9*)	38.2 (13.9)	37.6 (14.9)	<.001
Women, n (%)	*150* (*52.1*)	79 (69.9)	39 (60.9)	.001
Born in Canada, n (%)	268 (93.1)	104 (92)	60 (93.8)	.87
Married or living with someone in a marital-like relationship, n (%)	*179* (*62.2*)	44 (38.9)	26 (40.6)	<.001
Education (years), mean (SD)	*17.1* (*4*)	14.3 (3)	14.2 (3)	<.001
Working full-time in last year, n (%)	*184* (*63.8*)	34 (30.1)	16 (25)	<.001
With PROMIS^b^ *T* score >50, n (%)	173 (60.1)	75 (66.4)	*28* (*43.8*)	.02
Previously received a diagnosis of an anxiety disorder, n (%)	219 (76)	88 (77.9)	42 (65.6)	.17
Have received counseling or therapy from a professional for anxiety, n (% yes)	196 (68.1)	*95* (*84.1*)	43 (67.2)	.004
Was there a time when counseling or therapy from a professional for anxiety would have been helpful, but you did not receive it? n (% yes)	228 (79.2)	85 (75.2)	49 (76.6)	.67
Have received medication from a physician for anxiety, n (% yes)	207 (71.9)	*97* (*85.8*)	45 (70.3)	.005
Was there a time when medication from a physician for anxiety would have been helpful, but you did not receive it? n (% yes)	*170* (*59*)^b^	41 (36.3)	22 (34.4)	<.001

^a^Values in italics are significantly different from corresponding values in other samples.

^b^PROMIS: Patient-Reported Outcomes Measurement Information System.

### Information Importance

Table 2 provides the mean importance ratings given by those considering anxiety treatment in the future for 20 information topics concerning it. All 3 samples rated nearly all the information topics as *important* (mean ratings of ≥6). The mean ratings of importance and proportion of each sample that rated each topic as important were slightly higher in the clinic samples than in the self-help sample. In addition, the clinic samples rated the importance of information pertaining to the goal or outcome, common side effects, serious side effects, and advantages and disadvantages of treatment significantly more highly than members of the self-help sample.

**Table 2:** Ratings of the importance of information topics when considering the kinds of help available for anxiety problems.^a^

Information topic	Weighted mean rating	Self-help sample (n=288)		Psychology sample (n=113)		Psychiatry sample (n=64)	
		Mean rating (95% CI)	With a mean rating ≥6, n (%)	Mean rating (95% CI)	With a mean rating ≥6, n (%)	Mean rating (95% CI)	With a mean rating ≥6, n (%)
Effectiveness of treatment	6.6	6.4 (6.2-6.7)	219 (76.0)	6.9 (6.6-7.2)	74 (83.6)	6.8 (6.4-7.3)	54 (84.4)
How treatment works	6.6	*6.3* (*6.1-6.5*)	228 (79.2)	*7.0* (*6.7-7.3*)^b^	97 (85.8)	6.9 (6.5-7.3)	54 (84.4)
Advantages and disadvantages of treatment	6.6	*6.3* (*6.1-6.5*)^c^	216 (75.0)	7.0 (6.7-7.3)	94 (83.2)	6.9 (6.6-6.3)	53 (82.8)
What happens when treatment stops	6.5	6.3 (6.1-6.5)	213 (74.0)	6.8 (6.5-7.1)	90 (79.6)	6.9 (6.4-7.3)	53 (82.8)
Common side effects of treatment	6.5	*6.1* (*5.9-6.3*)^c^	193 (67.0)	7.0 (6.7-7.3)	98 (86.7)	7.1 (6.7-7.5)	55 (85.9)
Goal or outcome of treatment	6.5	*6.1* (*5.9-6.3*)^c^	181 (62.9)	7.1 (6.8-7.3)	101 (89.4)	6.8 (6.4-7.2)	53 (82.8)
How long treatment continues	6.3	6.1 (5.9-6.3)	205 (71.2)	6.6 (6.2-6.9)	87 (77.0)	6.6 (6.2-7.1)	51 (79.7)
All available treatments	6.3	6.1 (5.9-6.4)	202 (70.1)	6.7 (6.4-7.1)	85 (75.2)	6.4 (6.0-6.9)	45 (70.3)
Uncommon but serious side effects of treatment	6.3	*6.0* (*5.7-6.2)*^c^	216 (75.0)	6.6 (6.3-7.0)	87 (77.0)	6.8 (6.3-7.2)	52 (81.3)
How long it takes for treatment to produce results	6.2	*6.0* (*5.8-6.3*)	222 (77.0)	6.4 (6.0-6.7)	85 (75.2)	*6.7* (*6.3-7.1*)^b^	51 (79.7)
Cost of treatment to you	6.2	6.0 (5.7-6.2)	181 (62.8)	6.6 (6.2-7.0)	85 (75.2)	6.4 (5.8-7.0)	48 (75)
What you have to do as part of the treatment	6.2	6.0 (5.8-6.3)	205 (71.2)	6.5 (6.1-6.9)	87 (77.0)	6.5 (6.0-7.0)	50 (78.1)
Available counseling or psychological treatments	6.1	*5.8* (*5.6-6.0*)	184 (63.9)	*6.6* (*6.2-6.9*)^b^	83 (73.5)	6.5 (6.0-7.0)	45 (70.3)
Available medication treatments	5.7	5.6 (5.4, 5.8)	179 (62.2)	6.0 (5.6-6.4)	68 (60.2)	5.6 (5.0-6.2)	35 (54.5)
Self-help treatment	5.6	5.7 (5.5-5.9)	164 (56.9)	5.6 (5.1-6.0)	63 (55.8)	5.2 (4.7-5.8)	29 (45.3)
Exercise	5.6	5.6 (5.4-5.8)	156 (54.2)	5.6 (5.2-6.0)	62 (54.9)	5.3 (4.7-5.8)	28 (43.8)
Meditation	5.5	5.6 (5.4-5.8)	170 (59.0)	5.5 (5.0-5.9)	62 (54.9)	5.2 (4.6-5.8)	31 (48.4)
Herbal remedies	5.0	5.2 (4.9-5.4)	170 (59.0)	4.6 (4.1-5.1)	48 (42.5)	4.6 (3.6-5.0)	23 (35.9)
Cost of treatment to health care system	4.7	5.0 (4.7-5.3)	167 (58.0)	4.3 (3.8-4.9)	43 (38.1)	4.3 (3.6-5.0)	24 (37.5)
Marijuana	4.7	4.7 (4.4-5.0)	135 (46.9)	4.5 (3.9-5.0)	52 (46.0)	4.7 (4.0-4.4)	28 (43.8)

^a^Information was considered *important* if it received a mean rating of ≥6 on a scale ranging from 0 (*not important*) to 8 (*very important*). The second column provides the weighted average (across samples) of the ratings for each topic. Values in italics denote a CI that differs from the corresponding CI of one or both of the other samples.

^b^Denotes that the CI for a clinic sample is nonoverlapping with the CI of the self-help sample (at 2 decimal places).

^c^Denotes a CI for the self-help sample that is nonoverlapping with the CI of both clinic samples (at 2 decimal places).

### Information Received Among Those With Counseling or Therapy Experience

Table 3 provides ratings of the amount of information that respondents with psychological treatment experience had received when deciding to start this form of treatment for anxiety. Overall, the findings highlight that individuals in all 3 samples received, at best, a moderate amount of information on the different topics (all mean ratings ≤5.0). No topic received the highest rating, confirming that participants were generally dissatisfied with the amount of information they received. Thus, respondents in the self-help sample reported accessing significantly more information than those in the psychiatry sample on 9 of the 12 topics and significantly more information than the psychology sample on all the topics. Moreover, whereas the proportion of individuals in the self-help sample who felt that they had received an *appropriate* amount of information (rating≥6) ranged from 22% to 52% across topics, the proportion of individuals in the 2 clinical samples who felt this way was much lower, ranging from 2% to 27% across topics. All groups reported receiving the largest amount of information regarding available medication treatments, what the consumer has to do as part of the treatment, the goal or outcome of treatment, how treatment works, and how long treatment takes to produce results.

**Table 3:** Ratings of how appropriate the amount of information received was when making decisions about starting counseling or therapy for an anxiety problem.^a^

Information topic	Weighted mean rating	Self-help sample (n=194)		Psychology sample (n=94)		Psychiatry sample (n=41)	
		Mean rating (95% CI)	With a mean rating ≥6, n (%)	Mean rating (95% CI)	With a mean rating ≥6, n (%)	Mean rating (95% CI)	With a mean rating ≥6, n (%)
Available medication treatments	4.4	*5.0* (*4.7-5.2*)^b^	101 (52.1)	3.4 (2.9-3.8)	20 (21)	3.7 (3.3-4.2)	3 (7.3)
What you have to do as part of the treatment	4.3	*4.7* (*4.5-5.0*)^b^	80 (41.2)	3.8 (3.3-4.2)	25 (27)	3.6 (3.0-4.2)	7 (17.1)
Goal or outcome of treatment	4.3	*4.6* (*4.3-4.9*)	80 (41.2)	*3.8* (*3.4-4.2*)^c^	23 (25)	4.0 (3.4-4.6)	8 (19.5)
How treatment works	4.2	*4.6* (*4.3-4.8*)	64 (33.0)	*3.5* (*3.1-3.9*)^c^	19 (20)	3.8 (3.1-4.5)	7 (17.1)
How long it takes for treatment to produce results	4.0	*4.5* (*4.2-4.8*)	70 (36.1)	*3.1* (*2.7-3.5*)^c^	13 (14)	3.8 (3.1-4.4)	8 (19.5)
Effectiveness of treatment	4.0	*4.7* (*4.4-5.1*)^b^	87 (44.8)	2.9 (2.5-3.3)	14 (15)	3.2 (2.6-3.9)	3 (7.3)
Cost of treatment to you	3.9	*4.5* (*4.2-4.8*)^b^	74 (38.1)	3.1 (2.6-3.6)	21 (22)	3.2 (2.3-4.0)	8 (19.5)
How long treatment continues	3.8	*4.3* (*4.0-4.6*)^b^	43 (22.2)	*3.0* (*2.6-3.4*)^c^	14 (15)	3.3 (2.6-4.0)	6 (14.6)
Advantages and disadvantages of treatment	3.7	*4.4* (*4.1-4.7*)^b^	56 (28.9)	2.7 (2.3-3.1)	9 (10)	3.0 (2.3-3.8)	7 (17.1)
Common side effects of treatment	3.7	*4.3* (*4.0-4.6*)^b^	93 (47.9)	2.7 (2.3-3.2)	15 (16)	3.1 (2.4-3.8)	5 (12.2)
What happens when treatment stops	3.5	*4.2* (*3.8-4.5*)^b^	70 (36.1)	2.5 (2.1-2.9)	7 (8)	2.7 (1.9-3.5)	4 (9.8)
Cost of treatment to health care system	2.9	*3.8* (*3.4-4.2*)^b^	83 (42.8)	1.7 (1.3-2.2)	9 (10)	1.4 (0.77-2.0)	1 (2.4)

^a^Only participants who received previous psychological treatment for anxiety were included in the analyses. The weighted mean collapses across samples. The amount of information received was considered appropriate if it received a mean rating ≥6 on a scale with the following anchors: 0 (none), 2 (too little), 4 (moderate amount), 6 (quite a bit), and 8 (just right amount). Values in italics denote a CI that differs from the corresponding CI for one or both of the other samples.

^b^Denotes that the CI for the self-help sample is nonoverlapping with that of both clinic samples.

^c^Denotes a clinic sample CI that is nonoverlapping with the CI of the self-help sample.

### Information Received Among Those With Medication Experience

Table 4 provides ratings of the amount of information that respondents who had previously undergone medication treatment for anxiety had received when deciding to start this form of treatment. Once again, individuals in all 3 samples reported receiving, at best, a moderate amount of information on the different topics. None of the topics received a mean rating >4.8, suggesting that the participants were generally dissatisfied with the amount of information received. All groups reported receiving the greatest amount of information on how long it takes for treatment to produce results, the goal or outcome of treatment, what the consumer has to do as part of the treatment, how treatment works, and common side effects. Respondents in the self-help sample provided higher ratings than the other 2 groups regarding the amount of information available about counseling or psychological treatments, the cost to the consumer and the health care system, and treatment effectiveness.

**Table 4:** Ratings of how appropriate the amount of information received was when making decisions about starting medication for an anxiety problem.^a^

Information topic	Weighted mean rating	Self-help sample (n=204)		Psychology sample (n=94)		Psychiatry sample (n=43)	
		Mean rating (95% CI)	With a mean rating ≥6, n (%)	Mean rating (95% CI)	With a mean rating ≥6, n (%)	Mean rating (95% CI)	With a mean rating ≥6, n (%)
How long it takes for treatment to produce results	4.6	*4.8* (*4.6-5.1*)	102 (50)	*4.1* (*3.7-4.5*)^b^	32 (34)	4.5 (3.8-5.2)	13 (30.2)
Goal or outcome of treatment	4.4	*4.8* (*4.6-5.1*)	86 (42.2)	*3.8* (*3.3-4.2*)^b^	23 (24)	4.1 (3.5-4.7)	8 (18.6)
What you have to do as part of the treatment	4.2	4.4 (4.2-4.7)	53 (26)	3.8 (3.4-4.3)	24 (26)	3.8 (3.2-4.4)	11 (25.6)
How treatment works	4.1	*4.4* (*4.2-4.7*)	61 (29.9)	3.5 (3.1-3.9)	18 (19)	4.1 (3.4-4.8)	10 (23.3)
Common side effects of treatment	4.1	*4.4* (*4.2-4.7*)	67 (32.8)	*3.6* (*3.2-4.1*)^b^	23 (25)	3.7 (3.0-4.4)	11 (25.6)
Effectiveness of treatment	4.1	4.6 (4.3-4.8)	86 (42.2)	3.2 (2.8-3.6)	16 (17)	3.6 (2.8-4.3)^b^	7 (16.3)
Available counseling or psychological treatments	4.0	*4.5* (*4.2-4.7*)^c^	88 (43.1)	3.3 (2.9-3.8)	24 (26)	3.3 (2.6-3.9)	7 (16.3)
Advantages and disadvantages of treatment	4.0	*4.6* (*4.3-4.9*)	82 (40.2)	*2.8* (*2.3-3.2*)^b^	12 (13)	3.5 (2.8-4.3)	11 (25.6)
How long treatment continues	3.9	*4.3* (*4.0-4.6*)	49 (24)	*3.0* (*2.6-3.5*)^b^	18 (19)	3.7 (3.1-4.3)	7 (16.3)
Cost of treatment to you	3.8	*4.7* (*4.4-5.0*)^c^	80 (39.2)	2.5 (2.0-2.9)	9 (10)	2.5 (1.7-3.3)	6 (14)
Uncommon but serious side effects of treatment	3.8	*4.2* (*3.9-4.4*)	51 (25)	*3.1* (*2.6-3.5*)^b^	17 (18)	3.4 (2.7-4.1)	12 (27.9)
What happens when treatment stops	3.7	*4.3* (*4.0-4.6*)^c^	76 (37.3)	2.6 (2.1-3.1)	16 (17)	3.1 (2.3-3.8)	10 (23.3)
Cost of treatment to health care system	2.9	*3.9* (*3.6-4.3*)^c^	94 (46.1)	1.5 (1.1-1.9)	7 (7)	1.5 (0.8-2.1)	3 (7)

^a^Only participants who received previous medication treatment for anxiety were included in the analyses. The weighted mean collapses across samples. The amount of information received was considered appropriate if it received a mean rating≥6 on a scale with the following anchors: 0 (none), 2 (too little), 4 (moderate amount), 6 (quite a bit), and 8 (just right amount). Values in italics denote a CI that differs from the corresponding CI for one or both of the other samples.

^b^Denotes a clinic sample CI that is nonoverlapping with the CI of the self-help sample.

^c^Denotes that the CI for the self-help sample is nonoverlapping with that of both clinic samples.

### Amount of Information Received From Different Sources

Table 5 indicates the amount of information respondents received from different sources when considering anxiety treatment options. The internet was the highest-rated source, followed by family doctors. In line with the previously discussed findings, the self-help sample indicated receiving more information from 8 of the 10 sources than the clinic samples. Respondents in all samples indicated receiving the least amount of information from nurses.

**Table 5:** Ratings regarding the amount of information received from different sources.^a^

Information topic	Weighted mean rating	Self-help sample (n=286)		Psychology sample (n=113)			Psychiatry sample (n=64)	
		Mean rating (95% CI)	With a mean rating ≥6, n (%)	Mean rating (95% CI)	With a mean rating ≥6, n (%)	Mean rating (95% CI)		With a mean rating ≥6, n (%)
Internet	4.3	*4.7* (*4.5-5.0*)^b^	103 (36.0)	3.9 (3.4-4.4)	32 (28.3)	3.4 (2.9-4.0)		12 (18.8)
Family physician	3.6	3.6 (3.3-3.8)	74 (25.9)	3.8 (3.3-4.3)	33 (29.2)	3.6 (2.9-4.2)		16 (25)
Counselor or therapist	3.6	*4.3* (*4.0-4.6*)^b^	117 (40.9)	2.5 (2.0-2.9)	17 (15.0)	2.7 (2.0-3.4)		14 (21.9)
Psychiatrist	3.1	*3.7* (*3.4-4.1*)^b^	109 (38.1)	2.3 (1.9-2.8)	17 (15.0)	2.1 (1.4-2.7)		7 (10.9)
Friend	3.1	*3.6* (*3.3-3.9*)^b^	63 (22.0)	2.5 (2.0-3.0)	16 (14.2)	2.1 (1.5-2.6)		7 (10.9)
Psychologist	3.1	*3.7* (*3.3-4.0*)^b^	103 (36.0)	2.1 (1.7-2.6)	16 (14.2)	2.1 (1.4-2.8)		8 (12.5)
Book (eg, self-help book)	3.0	*3.6* (*3.3-3.9*)^b^	86 (30.1)	2.1 (1.7-2.6)	12 (10.6)	1.9 (1.3-2.5)		5 (7.8)
Family member (who is not a partner or spouse)	2.6	*2.9* (*2.6-3.1*)	49 (17.1)	2.3 (1.9-2.8)	14 (12.4)	*1.9* (*1.3-2.5*)^c^		6 (9.4)
Partner or spouse	2.3	*2.8* (*2.5-3.1*)^b^	49 (17.1)	1.5 (1.1-1.9)	8 (7.1)	1.4 (0.81-1.9)		5 (7.8)
Nurse	1.7	*2.1* (*1.8-2.3*)^b^	32 (11.2)	1.1 (0.73-1.5)	7 (6.2)	1.3 (0.71-1.9)		5 (7.8)

^a^The weighted mean collapses across samples. The amount of information received was considered *appropriate* if it received a mean rating ≥6. Values in italics denote a CI that differs from the corresponding CI for one or both of the other samples.

^b^Denotes that the CI for the self-help sample is nonoverlapping with that of both clinic samples.

^c^Denotes a clinic sample CI that is nonoverlapping with the CI of the self-help sample.

### Predictors of Information Importance and Information Received

Table 6 describes the regression analyses examining the predictors of the number of information topics considered *important* by participants (information importance composite score) and the number of topics for which an *appropriate* amount of information was received regarding either counseling or therapy or medication treatments (information received composite scores). The results are presented separately for the self-help sample and the combined clinical samples. The partial correlations (*pr*) reported in the table, when squared, indicate the unique proportion of the variance in a given outcome variable that is accounted for by each predictor when all other predictors and their shared variance have been accounted for in the relevant model.

In the self-help sample, gender and marital status were significant predictors of all 3 outcome variables. Men and married participants were more likely than women and unmarried participants (respectively) to rate a higher number of information topics as being important and feel that they had received an appropriate amount of information about both counseling or therapy and medication treatments after accounting for other predictors. Being born in (vs outside of) Canada emerged as an additional predictor of the number of topics found to be important, and both younger age and higher educational attainment emerged as additional predictors of how appropriate the amount of information received regarding medication treatment was found to be.

Gender was a less important predictor in the regressions performed on the combined clinic sample. Indeed, after accounting for other predictors, men were only more likely than women to report that they had received an appropriate amount of medication information. In contrast, years of education proved to be a somewhat more important predictor in the combined clinic (vs self-help) sample, with higher educational attainment predicting the number of topics found to be important and how appropriate the amount of information received regarding counseling or therapy (and, to a lesser extent, medication) was found to be.

**Table 6:** Predictors of composite scores for information importance, information received on counseling or therapy, and information received on medication for the self-help and combined clinic samples.^a^

Outcome variable				Clinic Sample (0=psychiatry, 1=psychology)		Gender (0=male, 1=female)		Birthplace (0=not Canada, 1=Canada)		Marital status (0=not married, 1=married)		Age		Years of education		Total PROMIS^b^ anxiety score		Anxiety disorder diagnosis		Therapy received or needed^c^		Meds received or needed^d^
**Self-help sample (n=283)**																						
	**Number of important topics**																					
		*Β^e^*	—^f^		−*1.42*		*4.28*		*2.3*		−0.003		−0.070		−0.020		.470		1.86		−2.20	
		*P* value	—		.*04*		*.002*		.*003*		.93		.42		.77		.67		.11		.08	
		*pr^g^*	—		−*0.130*		*0.180*		*0.180*		−0.010		−0.050		−0.020		0.030		0.100		−0.110	
	**Right amount of counseling or therapy information**																					
		*Β*	—		−*1.15*		1.11		*1.57*		−0.003		−0.070		−0.020		1.49		—		−0.450	
		*P* value	—		*.01*		.21		*.004*		.93		.42		.77		.12		—		.66	
		*pr*	—		−*0.180*		0.090		*0.200*		−0.160		0.270		0.180		0.110		—		−0.030	
	**Right amount of medication information**																					
		*Β*	—		−*2.11*		2.18		2.03		−*0.090*		*.310*		.090		−0.460		1.38		—	
		*P* value	—		*<.001*		.08		*.005*		*.007*		*<.001*		.09		.74		.28		—	
		*pr*	—		−*0.240*		0.120		*0.200*		−*0.020*		−*0.050*		0.290		−0.020		0.080		—	
**Combined clinic samples (n=170)**																						
	**Number of important topics**																					
		*Β*	−0.135		0.187		3.14		1.06		.040		*.295*		.050		−0.402		.035		2.65	
		*P* value	.88		.84		.07		.25		.21		*.05*		.47		.72		.98		.06	
		*pr*	−0.010		0.020		0.140		0.090		0.100		*0.150*		0.060		−0.030		0.002		0.150	
	**Right amount of counseling or therapy information**																					
		*Β*	.450		.020		−0.350		.120		−0.010		*.160*		.002		.450		—		.270	
		*P* value	.34		.97		.72		.80		.71		*.03*		.97		.49		—		.76	
		*pr*	0.080		0.003		−0.030		0.020		−0.030		*0.190*		0.004		0.060		—		0.030	
	**Right amount of medication information**																					
		*Β*	−0.117		−*2.26*		.360		1.40		−0.060		.249		−0.020		1.81		2.90		—	
		*P* value	.90		*.02*		.20		.13		.06		.08		.81		.13		.18		—	
		*pr*	−0.010		−*0.210*		0.020		0.130		−0.170		0.150		−0.020		0.130		0.120		—	

^a^The number of important topics was defined as the number of topics that received a rating of ≥6 for topic importance. The number of topics for which the right amount of counseling or therapy or medication information was provided equaled the number of topics receiving a rating of ≥6 for amount of information received. Values in italics are significant at *P*<.05.

^b^PROMIS: Patient-Reported Outcomes Measurement Information System.

^c^Therapy received or needed refers to the number of individuals who indicated that they had previously received counseling or therapy for anxiety in the past or who felt they would have benefited from doing so.

^d^Medication received or needed refers to the number of individuals who indicated that they had previously received medication for anxiety in the past or who felt they would have benefited from doing so.

^e^*Β*: unstandardized beta.

^f^Clinic sample membership is not applicable to the analyses within the self-help sample.

^g^*pr*: partial correlation.

## Discussion

### Principal Findings

This study addressed a gap in the literature in that it is one of the first to explore anxiety treatment information needs and one of the first to assess these needs in samples enrolled via different routes (ie, from psychology or psychiatry clinics vs on the web). Although individuals from both clinic and self-help samples are seeking information, we can speculate that they are likely at different points on their treatment-seeking journey. Specifically, whereas individuals in the clinic samples may have been actively engaging or preparing to engage in a specific form of treatment, individuals in the self-help sample may have still been seeking information about various treatment options, either for themselves or for another person (eg, family member or friend).

The 2 clinical samples comprised individuals with similar demographic characteristics. They also had a higher proportion of women than the self-help sample. This is congruous with the idea that women are more likely than men to seek treatment for mental health problems [13], even if men desire such information. The clinic samples were also significantly older than the self-help sample, which may simply reflect the fact that younger people are more regular users of the internet [14] and may therefore have been more likely to view our survey link posted on the ADAM website. Previous research also suggests that younger people are more likely to participate in shared decision-making [15], which may make them more interested in accessing information about topics such as anxiety and its treatment. Interestingly, most of the self-help sample reported a previous diagnosis of an anxiety disorder and had either received or felt they would have benefited from counseling or therapy *and/or* medication for anxiety in the past. This suggests that they may have been interested in participating in the survey because of their personal struggles regarding how best to manage symptoms of anxiety.

Not surprisingly, the psychology sample had the highest proportion of individuals with counseling or therapy experience. People who have previously received therapy may be more likely to continue to seek out therapy in the future, given that they tend to behave in a way consistent with their past behavior [16]. Interestingly, the psychology sample also had the highest proportion of individuals who had previously received medication treatment for anxiety. A possible explanation for this finding may be that the clinic from which the psychology sample was recruited has a long waitlist, and some individuals may have tried medication or another treatment for their anxiety before they were seen by this service. Finally, although more than three-quarters of individuals in each sample felt that they had not received counseling or therapy in the past when it might have been beneficial to do so, members of the self-help sample were more likely than those recruited through clinics to report feeling that they might have benefited from receiving medication in the past. This supports the view expressed earlier in the Discussion section that members of the self-help group were more ambivalent about what the best course of treatment for anxiety is likely to be. Overall, the sample differences described above support the view that people currently seeking *treatment* differ from those currently seeking *information* in terms of their information needs.

All 3 samples viewed information on a wide range of topics as important, consistent with our group’s earlier work involving information needs related to stress, anxiety, and depression [7,9]. This is also consistent with the information needs for people with other health issues such as cancer [17]. The ratings of the importance of specific information topics in this study were also similar to those in our earlier research. The mean ratings of topic importance and proportion of individuals who rated a topic as important were slightly higher in the clinic samples than in the self-help sample. These findings may be related to the fact that those in the clinic samples were actively seeking or engaged in a course of treatment. In their *Stages of Change* model, Prochaska and DiClemente [18] outlined several stages of behavior change, including *precontemplation* (more than 6 months until intended action), *contemplation* (action in the next 6 months), *preparation* (action in the next month), *action* (action begins), *maintenance* (at least 6 months into an action), and *termination* (during which an individual will not return to old habits). As suggested above, the clinic samples may be quite far along in this process, having made a decision to seek treatment (preparation) and having met with health care providers, such as family physicians, to obtain a clinic referral (action). The discussions that they may have had with health care providers may have helped them reflect on the kind of information they would want concerning anxiety treatment. In contrast, members of the self-help sample may primarily be at the precontemplation or contemplation stages and be focused on finding general information for themselves or on behalf of another person.

An area unique to this study was the examination of the amount of information previously received. Overall, the self-help sample provided higher ratings regarding the amount of information received when considering starting counseling or therapy or medication treatment for anxiety. The fact that the psychology clinic sample reported greater treatment experience than the self-help group and that both clinic samples had likely had more opportunities to speak with health care providers about anxiety treatment may have meant that these groups had a better sense of whether they had received *the right* amount of information on the different topics at the time of the survey, compared with the self-help group. If so, this would suggest that despite seeing a mental health treatment provider, these individuals may still feel inadequately informed about treatment options. In contrast, if the self-help sample was at an earlier stage of the information gathering or treatment-seeking process, they may have (1) been less certain about how much information was actually available on certain topics, (2) had less need for information, or (3) sought information from fewer sources. These factors, alone or in combination, might have led them to feel more satisfied with the amount of information they received. In either case, it is important to note that none of the groups provided high ratings for the appropriateness of the amount of information they received.

It is noteworthy that the clinic samples did not report feeling adequately informed about medical treatments if they were currently seeking counseling or therapy or vice versa. This suggests that people are not necessarily given a choice when starting a treatment, despite the efficacy of both therapy and medication in treating anxiety problems [19-21]. An important clinical implication of these findings is that health care providers need to have more in-depth discussions with their patients about a broad range of topics, including different treatment options, to help their patients decide on the best course of action.

Respondents in the self-help sample indicated that they had received more information from a range of different sources than the clinic samples. Again, this might suggest that members of the self-help group are interested in gathering much information, whereas the clinic samples are at a stage where they have a better idea of the type of information they want or need and where to obtain it. We also found that in the self-help sample, being a man and being married positively predicted the number of information topics rated as important and the appropriateness of the amount of counseling or therapy and medication treatment information received. Men in this sample may have had less treatment experience than women, which could have influenced their ratings in these areas. It may also be that married people are often interested in gathering information to help them understand or support a spouse who is struggling with anxiety, rather than for themselves. In such cases, it may be useful to include the spouse in an initial treatment session designed to provide psychoeducation about anxiety and its treatment.

All 3 study groups reported that their family physicians were important sources of information. This is not surprising given that the family physician is likely to be one of the first health care providers one sees when struggling with mental health problems such as anxiety. However, it does speak of the importance of family physicians engaging in continuing education to ensure that the information they provide is current and that they can address their patients’ questions. Given the range of topics identified as being of interest, one can imagine how difficult it would be to review all of these topics in a typical primary care visit of 10 to 15 minutes or even in a specialist visit of 20 to 50 minutes. More importantly, from the patient’s perspective, it would be very challenging to process and remember large amounts of information if presented orally, especially for those struggling with anxiety. For these reasons, it would be helpful for health care providers to deliver information in the form of patient-oriented brochures or web-based information that can be reviewed over a longer period and revisited as needed [3,5,8]. A limitation of paper-based formats is that it would take considerable space to address all the topics identified as important in this study and to provide context regarding the quality of the available scientific evidence. An advantage of websites is that they can incorporate drop-down menus and links that allow the consumer to obtain more detailed information about topics of interest. However, research by our group suggests that internet sources are of variable quality [22]. This is of concern, given that all 3 groups in this study reported that the internet was an important source of information. More effort should also be made to ensure that high-quality print and web-based resources are available. Ensuring that the reading level of these materials is low and the clarity of the writing is high is important to ensure that less well-educated members of the public can process them.

### Limitations

Although this study addresses gaps in the literature by assessing what information people view as important in considering help for anxiety and what anxiety information they have received in the past, it is not without limitations. A limitation of this study is that it examined the objectives from a quantitative methods perspective. Other useful information may be obtained by collecting open-ended responses in semistructured interviews and using a qualitative approach to data analysis. Another limitation is that this sample may not generalize to individuals not seeking help or information, as many individuals with anxiety do not seek help [23]. A third limitation is that participants in the clinic and self-help samples were individuals in the process of seeking help or information; therefore, the generalizability of the results to all individuals with anxiety problems may be limited. Furthermore, the clinic participants who responded to the survey may not truly reflect those seeking treatment within each clinic. Compared with those who did not participate, those who did were likely individuals who desired more information and aimed to engage in various methods of obtaining information, such as completing a survey related to anxiety treatment. Another limitation of the information needs questions is that they did not undergo a series of reliability and validity tests. This could be a subject of future research. Finally, we were unable to determine the response rate for the self-help sample. Some individuals may have clicked on the link to the survey but decided not to complete it. This is an issue because it is not possible to determine how the sample of respondents compared with the total population of those visiting the website.

### Implications

These results indicate that people are interested in a wide range of information topics on anxiety treatment. This is similar to the information needs for people with other health issues such as cancer [17]. However, individuals often do not receive the amount of information that they desire. Health care providers’ understanding of information needs and preferences for persons with anxiety (and other health problems) provides a better appreciation of the patient-preferred role in the treatment decision-making process. This study also demonstrated differences in preferences for the amount of information among individuals. A way to deal with such differences is to produce information focused on each topic and allow consumers to choose their areas of interest. Our research group is currently developing evidence-based materials to treat anxiety. Another issue raised by the findings of this study is that persons with anxiety may not be provided information on different treatment options when seeking treatment. This leads to the question of whether there are any barriers to discussing different options with their health care providers. A hypothesis is that there is a lack of high-quality, evidence-based information that can be used by consumers and health care providers to allow consumers to make informed decisions. Another hypothesis is that health care provider knowledge may vary across providers. For example, general health care providers may not have the in-depth knowledge of treatment options that specialists do [24]. Overall, increasing public knowledge and the use of health information results in more positive attitudes toward help-seeking [2].

### Conclusions

This study fills an important gap in the literature by examining the information needs of people with anxiety. The results suggest that people with anxiety are interested in information developed to answer important questions concerning anxiety treatment. Information needs for other common mental health problems have been found to be similar [7,9,25]. Of particular interest to consumers is information about treatment goals and effectiveness and what happens when the treatment stops [7,9,25]. The wide range of topics judged to be important by individuals with anxiety suggests that it would be very difficult to address these information needs via oral communication during health care visits or using currently available materials. Therefore, it is imperative that high-quality, evidence-based resources be created to assist individuals in making decisions about treatment for problems with anxiety.

## Abbreviations

ADAM: Anxiety Disorders Association of Manitoba
PROMIS: Patient-Reported Outcomes Measurement Information System
